# From individuals to communities: How genomics is transforming biodiversity conservation

**DOI:** 10.1590/1678-4685-GMB-2025-0329

**Published:** 2026-07-27

**Authors:** Larissa S. Arantes, Maycon Douglas de Oliveira, Henry Paul Granger-Neto, Zandora Celeste Hastenreiter, Rafael A. Baggio, Barbara Regina Neves Chaves, Fabrício Rodrigues Santos

**Affiliations:** 1 Universidade Federal de Minas Gerais, Instituto de Ciências Biológicas, Departamento de Genética, Ecologia e Evolução, Belo Horizonte, MG, Brazil.; 2 Universidade Federal de Minas Gerais, Instituto de Ciências Biológicas, Programa de Pós-Graduação em Ecologia, Conservação e Manejo da Vida Silvestre, Belo Horizonte, MG, Brazil.; 3 Universidade Federal de Minas Gerais, Centro de Microscopia, Belo Horizonte, MG, Brazil.

**Keywords:** Reference genomes, chromosome-level genome, comparative genomics, population genomics, environmental genomics

## Abstract

Genomics has rapidly become one of the most transformative scientific approaches in modern conservation biology. As biodiversity faces increasing threats from climate change, habitat degradation, invasive species, and other human impacts, the use of genomics is providing conservation scientists with unprecedented tools to monitor biodiversity, manage endangered species, and anticipate ecological challenges. This review synthesizes recent advances in genomic applications for wildlife conservation, focusing on high-resolution whole-genome sequencing, comparative genomics, population genomics, local adaptation and environmental DNA (eDNA). The review concludes with a forward-looking discussion on integrating genomics, biodiversity monitoring, and conservation practices into a new era of comprehensive and continuous surveillance of threatened populations across diverse ecosystems.

## Introduction

Biodiversity loss represents one of the most critical challenges of the 21^st^ century, with extinction rates accelerating under the increasing impact of climate change, habitat destruction, overexploitation, introduced species, and other human impacts. Conservation biology, a field traditionally reliant on ecological and demographic data, has largely turned to genetic data as a means of addressing these challenges with greater precision and scalability (Allendorf et al., 2010; Shafer et al., 2015).

The advent of genomics in the last decade of the 20^th^ century opened unprecedented opportunities to understand species biology at the molecular level, assess spatiotemporal genetic diversity, identify cryptic species, reconstruct demographic histories, understand adaptation patterns, characterize community diversity, comprehend ecological associations, and thereby advance the field of biodiversity conservation. The rapid decline in sequencing costs, the development of new high-throughput and portable sequencing technologies, and advances in bioinformatics tools have further expanded the accessibility and applications of genomics in both laboratory and fieldwork settings. Genomic approaches now enable the investigation of questions that were previously intractable with traditional genetic tools - particularly those linking genomic variation to individual fitness, local adaptation, and evolutionary potential (Allendorf et al., 2010). Together, such genetic insights enable more targeted and precise interventions for species recovery and ecosystem balance.

Small and declining populations are experiencing a series of detrimental genetic changes, including the loss of genome-wide diversity, increased inbreeding and accumulation of deleterious mutations - collectively referred to as genomic erosion (Díez-del-Molino et al., 2018). With recent advances in genomic technologies, it has become possible to detect and quantify genetic erosion signatures - such as indicators of inbreeding, genetic drift, demographic instability, population fragmentation, and purifying selection - thereby enabling the precise identification of negative impacts in endangered populations, including inbreeding and outbreeding depression, the expression of deleterious alleles, maladaptation, and reduced adaptive potential (Leroy et al., 2018). Importantly, the ability to quantify genetic erosion at a genomic level provides conservationists with an objective and quantifiable metric to assess species extinction risk (Bosse and van Loon, 2022).

Beyond improved precision and efficiency, the new genomic approaches offer the unique advantage of distinguishing between neutral diversity (primarily shaped by population size and gene flow) and adaptive diversity (mainly driven by selection), thereby allowing management strategies to focus on maintaining the adaptive genetic potential of species (Leroy et al., 2018). These advances also enable the identification of the genetic underpinnings of individual fitness, local adaptation to environments, and lineage-specific evolutionary trajectories. The recent availability of thousands of high-quality reference genomes spanning the whole tree of life has further facilitated these discoveries by providing the comparative framework necessary to link genomic variation with functional traits and evolutionary processes across species (Grueber, 2015). 

Genomics has also revolutionized the way biodiversity is assessed and monitored in natural ecosystems. Environmental DNA (eDNA) analyses enable the detection of species’ presence without the need for direct observation, offering major advantages when studying endangered, invasive, or cryptic taxa. Metabarcoding and metagenomic approaches now enable the examination of entire communities and ecological associations through non-invasive sampling of environmental substrates such as soil, water, and air (Bohmann et al., 2014; Barnes and Turner, 2016; Çevik and Çevik, 2025). This allows the assessment of the spatial distribution and temporal dynamics of species and their ecological networks. From an ecological perspective, eDNA analyses enable the evaluation of richness, dynamics, connectivity, and temporal changes within communities. Near-real-time monitoring and assessment through these approaches are key to conserving biodiversity in a world undergoing rapid and drastic anthropogenic environmental change (Thomsen and Willerslev, 2015). 

This review provides a comprehensive survey of technological and analytical advances, applications, and case studies of conservation genomics, with an emphasis on current tools and future perspectives. Here, we also explore how genomic data can inform conservation decisions by offering insights beyond the reach of traditional genetic tools. Our focus is on key applications such as genome erosion measures, identifying evolutionary adaptation, comparative genomics, and community monitoring using eDNA.

## Advances in genomic approaches for biodiversity conservation 

A wide array of genomic methods is now available for investigating conservation-related questions, each varying in genomic coverage, from single-locus markers to chromosome-level genome assemblies. The selection of an appropriate approach depends on multiple factors, including genome size, available resources, sampling, and specific research objectives (Christiansen et al., 2021). For instance, the number of genetic markers or single nucleotide polymorphisms (SNPs) required to resolve relationships among individuals, populations or species depend largely on the informative variation degree (Peterson et al., 2012); while studies focused on association mapping or the detection of selection signatures rely on genome-wide patterns of linkage disequilibrium, which are in turn also shaped by demographic history (Lasky et al., 2022).

Population genomic tools such as reduced representation sequencing (RRS) techniques (i.e., Restriction site-Associated DNA sequencing - RAD-seq, Genotyping by Sequencing - GBS), SNP arrays, and low-coverage whole-genome resequencing have become indispensable for elucidating population-level processes. These methods allow for the inference of effective population sizes (N_e_), genetic bottlenecks, migration rates, and putative adaptive variation (Andrews et al., 2016). For example, SNPs identified by GBS allowed characterization of the evolutionary dynamics of the four remaining Brazilian merganser populations (Campos et al., 2023). Notably, population genomic studies usually provide a greater inferential power from sampling larger numbers of individuals with wider geographic sampling coverage, rather than maximizing the number of genetic markers (Christiansen et al., 2021). Consequently, researchers often prioritize cost-effective approaches that provide sufficient genomic resolution to address population-level questions while maximizing sample size and statistical robustness.

One of the most important advances in conservation biology in the 21^st^ century has been the rise of whole-genome sequencing (WGS) of complex genomes. Besides enabling surveys of genome-wide SNPs, WGS allows the characterization of a wide diversity of structural and sequence variations and their spatial association with functional or regulatory elements across the genome, thereby increasing the detection power of selection signatures, and enabling the identification of genotypes directly associated to phenotypic traits (Fuentes-Pardo and Ruzzante, 2017).

Methodological advances in the last decade have also enabled the generation of high-quality reference genomes with contiguous and phased chromosomes, showing accurate gene order and structural differences. Together with the functional annotation of the genome, conservation scientists now have the resources needed to investigate the full breadth of genetic variation across coding and non-coding regions, to assess adaptive loci and structural features, and to explore evolutionary processes in unprecedented detail (Formenti et al., 2022). These advances rely on a combination of techniques, including single-molecule long-read sequencing for contig assembly (i.e., PacBio Single Molecule Real-Time sequencing - SMRT, Oxford Nanopore Technologies sequencing - ONT), followed by scaffolding with linked reads, optical maps, and/or high-throughput sequencing Hi-C methods (Rhie et al., 2021). Recent efforts are focusing on generating telomere-to-telomere (T2T) genomes to fill the remaining gaps (∼5%-10% missing sequence information) in reference assemblies (Murphy and Harris, 2024).

Several global collaborative initiatives are generating reference genomes for thousands of eukaryotic species, creating foundational datasets for conservation genomics (Lewin et al., 2018). Some consortia are taxon-focused, such as the Vertebrate Genomes Project, Ten Thousand Plant Genomes Project, and 1000 Fungal Genomes Project. Others are regionally oriented, including the Africa BioGenome Project (Ebenezer et al., 2022), European Reference Genome Atlas (Mc Cartney et al., 2024), Genotropics (genotropics.org), and the Genomics of the Brazilian Biodiversity (Vilaça et al., 2024). In parallel, broader umbrella initiatives, such as the Earth BioGenome Project (Lewin *et al*., 2018), aim to integrate and coordinate some efforts globally. These worldwide networks of researchers are not only creating valuable resources but also standardizing sequencing and assembly methods, ensuring comparability across taxa. In conservation contexts, reference genomes allow finer population surveys using resequencing approaches with precise mapping of SNPs, and many other detailed analyses that were precluded with former low-quality (draft) genomes. Such approaches exemplify how genomics can be embedded into broader conservation frameworks, supporting both ecological sustainability and knowledge dissemination worldwide.

We underscore that translating reference genomes (and other genomic resources) into tangible conservation outcomes requires additional steps. While reference genomes are valuable scientific tools, their production alone rarely results in effective conservation actions without subsequent population-level analyses or applied interventions. Furthermore, a single genome cannot represent the genetic diversity of an entire species (Pegueroles et al., 2024). Thus, the future of conservation genomics will probably focus on integrating reference genomes and WGS data across multiple individuals (pangenomes). Notable examples of conservation studies that go beyond single reference genomes include research on Tasmanian devils (Brandies et al., 2019) and fin whales (Nigenda-Morales et al., 2023).

At the community level, genomics has also revolutionized our understanding of species distributions, community composition, and their spatial and temporal dynamics. The metabarcoding approach combines polymerase chain reaction (PCR) amplification and next-generation sequencing (NGS) of standardized genetic markers (barcodes) from mixed DNA fragments, followed by bioinformatic pipelines for the identification of one or multiple taxonomic groups within a community (Ruppert et al., 2019). In contrast, metagenomics employs shotgun sequencing to recover a large proportion of the DNA present in an environmental sample, thereby overcoming PCR-related limitations such as primer bias, contamination, and amplification errors. This approach enables a more comprehensive characterization of biological communities, encompassing species composition, functional gene annotation, metabolic pathways, and the reconstruction of partial or even complete genomes (Quince et al., 2017). Both methods can be applied to a wide variety of samples, including DNA extracted from water, soil, and air (environmental DNA, or eDNA), as well as from organism-derived materials such as digestive contents, feces, pollen, or seeds. These approaches enable community-level characterization and provide insights into ecological interactions among species, all through non-invasive sampling (Bohmann et al., 2014; Cristescu and Hebert, 2018).

## Inbreeding and outbreeding issues

Driven by recent anthropogenic changes in the environment, many populations are undergoing genetic erosion, characterized by the loss of advantageous alleles and adaptive genotype combinations (Leroy et al., 2018). Declining populations usually experience intensified genetic drift and inbreeding, which together erode standing genetic variation and reduce the population’s adaptive potential. Inbreeding (mating among relatives) generally causes offspring to have reduced fitness, a phenomenon known as inbreeding depression, arising from increased homozygosity that exposes deleterious recessive alleles, or from decreased heterozygosity at loci exhibiting heterozygous advantage (Charlesworth and Willis, 2009; Kardos et al., 2016). 

High-resolution genomic data have transformed the study of inbreeding by enabling direct detection of genomic regions that are identical-by-descent (IBD), which are inherited from a common ancestor without recombination (Curik et al., 2014). Inbreeding increases the occurrence of IBD segments throughout the genome, as related individuals are likely to share many identical chromosome segments (haplotypes) inherited from their common ancestors (Kardos et al., 2016). The size and distribution of IBD segments across the genome provide insights into the history and degree of inbreeding: long IBD segments typically result from recent common ancestors, whereas shorter segments reflect a more distant shared ancestry due to accumulated recombination events over generations (Curik *et al*., 2014; Kardos *et al*., 2016).

**Figure 1-:**
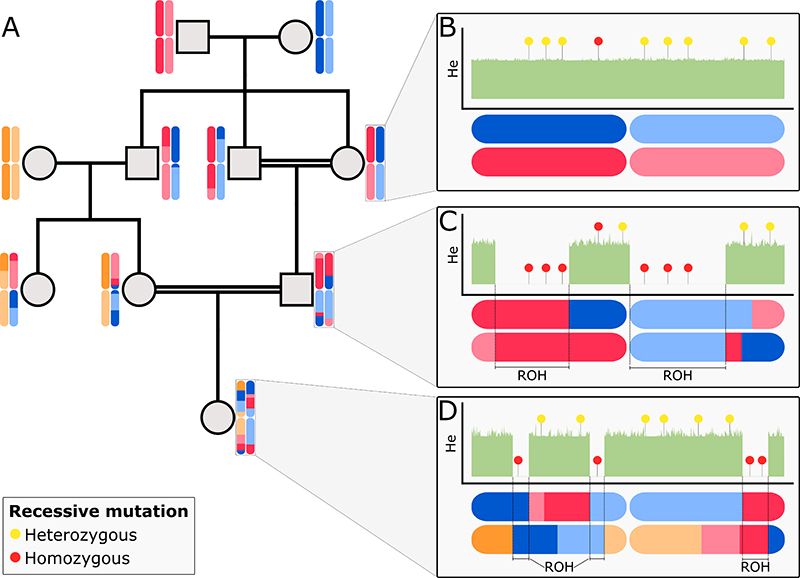
Genomic variability consequences of inbreeding as revealed by Runs of Homozygosity (ROH). A) An example of pedigree illustrating inbreeding. B) Expected heterozygosity (He) pattern in an offspring from an outbred mating, showing homogeneous heterozygosity across a chromosome. C) The effect of inbreeding between closely related individuals: long, contiguous ROH segments are present. These regions exhibit reduced heterozygosity and can lead to the homozygosity of deleterious recessive mutations, increasing genetic load. D) The effect of older/more distant inbreeding: ROH segments are fragmented due to the crossing-over recombination across generations, leading to shorter ROHs.

Whole-genome data has allowed the identification of IBD chromosome segments as stretches of homozygous genotypes, known as runs of homozygosity (ROH), which serve as a measurement of individual inbreeding (Figure 1). In an outbred mating, we expect homogeneous levels of heterozygosity across the whole genome (Figure 1B). Inbred genomes resulting from the crossing of closely related individuals often produce large ROHs (Figure 1C), which are more likely to have recessive homozygous alleles, frequently deleterious, and an increased genetic load (Szpiech et al., 2013). Likewise, across generations, IBD segments are fragmented due to the crossing-over recombination, leading to shorter ROHs (Figure 1D). 

ROH-based analyses can estimate the timing of inbreeding events by converting ROH length into the expected number of generations since the individual’s parents shared a common ancestor, assuming an average recombination rate (Kardos et al., 2018; Saremi et al., 2019). For example, ROH analysis revealed a peak of inbreeding in the scimitar-horned oryx when the species was close to extinction in the wild (Humble et al., 2023), demonstrating the power of ROH to infer the strength and timing of recent bottlenecks and to contextualize contemporary patterns of nucleotide diversity within a historical framework.

Uncovering the genomic basis of inbreeding depression has long been a central goal in conservation biology, and recent advances in genomic resolution have now made this possible. For example, genetic studies have demonstrated the association between strongly deleterious recessive mutations and severe inbreeding depression in gray wolves of Isle Royale (Robinson et al., 2019) and killer whales (Kardos et al., 2023). Several studies have shown that ROH analysis can also be used to directly estimate inbreeding depression (Shafer and Kardos, 2025). Indeed, deleterious alleles contributing to inbreeding depression are usually associated with chromosome regions containing fewer ROH (Hendricks et al., 2018). Furthermore, new ROH-based genomic statistics were proposed to predict the risk of inbreeding depression across species, offering a valuable tool for conservation (Kyriazis et al., 2025). 

Advances in genomics have also made it possible to evaluate the potential negative impact that inbreeding will have on population fitness relative to an optimal genotype of an outbred population, referred to as genetic load (Dobzhansky 1957; Bosse and van Loon 2022). It can be partitioned into two main components: the realized (or expressed) load, which reflects the loss of fitness in the current generation due to deleterious alleles currently expressed in the population, and the masked (or inbreeding) load, which captures the potential fitness decline caused by (partially) recessive deleterious mutations that may become expressed in future generations through inbreeding or demographic changes (Bertorelle et al., 2022). Together, these components constitute the total genetic load, encompassing both present and potential future impacts of deleterious variation on population viability.

Population genetic theory predicts two contrasting outcomes for genetic load in small populations (von Seth et al., 2022). On one hand, strong genetic drift and weakened purifying selection can lead to the accumulation of deleterious mutations. This pattern is observed in the crested ibis, which experienced a severe population bottleneck resulting in high levels of inbreeding and an increased deleterious mutation load in the current population (Feng et al., 2019). On the other hand, under certain conditions - such as gradual population decline or long-term isolation with small population size, or intensified selection against recessive harmful alleles - purifying selection can reduce recessive deleterious alleles, resulting in the purging of genetic load (von Seth *et al*., 2022). Such purging effects have been reported in Indian tigers (Khan et al., 2021), Arabian leopard (Mochales-Riaño et al., 2023) and three-toed maned sloths (Arantes et al., 2025b).

Genomic knowledge about genetic load and ROH has been increasingly applied in practical conservation efforts. Genetic rescue, the artificial (re)introduction of new or rare genetic variants into a population, can benefit from genomic information for designing strategies aiming to reduce inbreeding depression and to increase genetic variation and population viability. For example, ROH insights can inform conservation managers about the selection of individuals for introduction or translocation by identifying those with minimal shared ROH, thereby maximizing the genetic benefits of rescue efforts (Saremi et al., 2019).

Genomics-informed breeding has become an essential tool for managing captive populations of threatened species, allowing conservationists to identify optimal pairings that maintain genetic diversity and adaptive potential, and minimize the accumulation of deleterious alleles, ultimately improving the long-term viability of populations destined for reintroduction or long-term captivity (Segelbacher et al., 2022). For example, in the critically endangered pink pigeons, *in silico* genomic simulations have been applied to predict breeding pairs that would produce offspring with a lower realized genetic load than random matings, providing a promising framework for reducing genetic risks in captive populations (Speak et al., 2024). Similarly, genomic data for about 50 captive individuals of the critically endangered Brazilian merganser were used to guide pairings to reduce inbreeding and the occurrence of a potentially deleterious pigmentation phenotype (Campos et al., 2024).

The crossing of individuals from genetically distinct populations may eventually lead to reduced fitness in their offspring, a phenomenon known as outbreeding depression. This occurs due to the introduction of maladaptive alleles into populations locally adapted to different environmental conditions, or the breakdown of coadapted gene complexes (Edmands 2007). It is particularly common in captivity management or during translocation or reintroduction into the wild because conspecific individuals from different localities and environments are more likely to interbreed. Indeed, this phenomenon is related to the formation of the first reproductive barriers in the speciation process. Thus, divergent populations with incipient reproductive barriers shaped by natural selection are especially prone to outbreeding depression (Rollinson et al., 2014).

Recent research underscores the need to carefully balance the benefits of genetic rescue with the risks of outbreeding depression in conservation translocations. For example, Walsh et al. (2024)  identified divergent regions associated with pigmentation, immune response, and food intake between red grouse populations in Great Britain and Ireland, cautioning against translocations that could swamp locally adapted alleles or introduce maladapted genotypes. In contrast, Reid et al. (2025) used comprehensive genomic metrics - including heterozygosity, ROHs, N_e_, genetic differentiation, genetic load, and adaptive and structural variation - to support assisted gene flow between carefully selected donor and recipient populations.

Despite the relative importance of inbreeding and outbreeding depression in *ex situ* and *in situ* conservation management, practical efforts must account for a diverse set of crucial indicators of the conservation status and genomic health of a given species (or population) to translate all this knowledge into effective action (Shafer et al., 2015, Holderegger et al., 2019).

## Genomic metrics applied to conservation 

**Figure 2-A):**
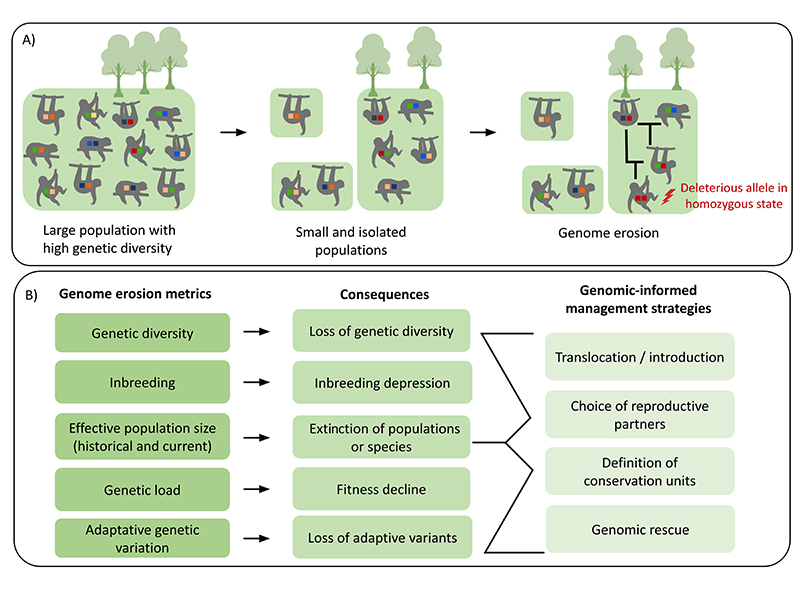
Schematic representation of genomic erosion in small and isolated populations. The original large population (left) exhibits high genetic diversity, illustrated by multiple alleles at a given locus (colored squares). Following habitat fragmentation, populations become geographically isolated, leading to altered allele frequencies and the loss of alleles. In these small, isolated populations, inbreeding increases and previously masked deleterious mutations are expressed in homozygous state, resulting in reduced individual fitness and accelerated genomic erosion. B) Recent advances in genomic technologies made it possible to detect and quantify genetic erosion signatures and their biological consequences, including inbreeding depression, the fitness decline, and loss of adaptive potential. The final goal is to guide the management and recovery of threatened populations and species. In the figure, the bracket from “Consequences” to “Genomic-informed management strategies” signifies a many-to-many relationship, i.e. that multiple consequences are linked to multiple management strategies.

Genetic information provides essential insights that guide the management and recovery of threatened populations and species. The estimation of key population parameters, including N_e_ and levels of genetic diversity, inbreeding and genetic load, captures fundamental evolutionary processes - such as gene flow, adaptation, demography and fragmentation - that shape the long-term viability and adaptive potential of species (Willi et al., 2022). Furthermore, genetic data have illuminated many aspects of reproductive behavior, social structure, and life history. By integrating these indicators into management planning, conservation practitioners can identify actions aimed at increasing population sizes, genetic variation and evolutionary potential, mitigating genetic load and maintaining genetic distinctiveness (Figure 2).

Recent initiatives have proposed a standardized framework that harmonizes genetic metrics to track biodiversity change across regions and timescales. For example, the Group on Earth Observations-Biodiversity Observation Network (GEO BON) proposed four genetic Essential Biodiversity Variables (EBVs) - genetic diversity, genetic differentiation, inbreeding, and N_e_ - to serve as measurable indices for monitoring conservation status and trajectories (Hoban et al., 2022). These Genetic EBVs offer quantitative measures that make the assessment of genetic status more transparent and comparable across taxa. Ultimately, the goal is to integrate these metrics into international monitoring schemes to provide policymakers with evidence-based tools for prioritizing conservation actions.

Several empirical studies have suggested that genetic metrics such as heterozygosity, Watterson’s theta, autozygosity and N_e_ are usually associated with species’ conservation threat status (Zoonomia Consortium, 2020; DeWoody et al., 2021; Jeon et al., 2024). These findings highlight the potential of genomic data to enhance the classification of threat levels and establish baselines for long-term monitoring of population health and viability. Together, the development of Genetic EBVs, complementary indicators, and empirical demonstrations of their predictive value highlight the importance of embedding genetics into biodiversity monitoring, conservation assessments, and policy decision-making. However, the threat classification system employed by the International Union for Conservation of Nature (IUCN) fails to incorporate many important genetic parameters (Jeon *et al*., 2024), though there exists an IUCN Species Survival Commission Conservation Genomics Specialist Group focused on addressing this very issue (https://www.cgsg.uni-freiburg.de/).

Incorporating genomic proxies into extinction assessments remains challenging, largely because of the complexity of genetic erosion. Small and geographically isolated populations are generally expected to be highly susceptible to extinction due to declining heterozygosity, elevated inbreeding levels, accumulation of harmful mutations, and the consequent loss of adaptive potential. However, some studies have reported exceptions to these expectations. Some populations exhibit limited inbreeding or low levels of deleterious mutations due to high selection pressure that purged strongly deleterious recessive alleles in previous generations (Robinson et al., 2019). Most likely, species-specific life-history traits and past population bottlenecks influence these patterns, potentially overshadowing the genetic consequences of recent demographic declines (Díez-del-Molino et al., 2018). Thus, the use of genetic erosion metrics in conservation requires consensus on appropriate techniques and threshold values to bridge the gap between scientific research and practical conservation efforts (Shafer et al., 2015; Holderegger et al., 2019; Bosse and van Loon, 2022). 

The power of genomics also resides in the fact that some genetic health proxies can be estimated from a high-resolution genome of a single individual. For example, Zoonomia Consortium (2020) analyzed one individual from 240 mammalian species and found that more threatened species tend to have lower heterozygosity and higher homozygosity. Demographic histories reconstruction done for one specimen of each sea turtle species shed light on past climate-driven contractions and expansions, offering insights into species resilience and vulnerability under predicted future climate scenarios (Arantes et al., 2025a). It is important to note that species-wide patterns may diverge from estimates based on single individuals, as they are further shaped by social structure, ecological dynamics, behavioral traits, reproductive idiosyncrasies, and environmental pressures that must be accounted for to achieve a comprehensive assessment.

Population genomics has been also used to study connectivity among fragmented populations, a critical consideration for species whose original ranges are increasingly divided by anthropogenic impact (Hohenlohe et al., 2021). For instance, genomic studies of Brazilian merganser have revealed three distinct population clusters, with direct implications for recovery strategies (Campos et al., 2023). The high resolution afforded by whole-genome data can uncover subtle population substructure that may be missed using traditional markers. This was demonstrated in genomic studies of great apes, where the subspecies *Pan troglodytes ellioti* was only identified through genome-wide SNP data, remaining undetected in analyses based on microsatellites or limited SNP datasets (Bowden et al., 2012).

Incorporating all available genomic information when designing and planning management strategies is essential for understanding the potential biological and ecological consequences of altering the genetic composition of a species or population. Such alterations could negatively impact conservation efforts, particularly by disrupting locally adapted allele networks.

## Natural selection and adaptation

Characterizing the impact of natural selection on the conservation of wild populations presents a formidable challenge (Lässig et al., 2017). Selection should always be considered in the response of a given population/species to biological and ecological variables, such as climate, geographical features, biotic interactions and elevation. However, only the effect of genetic drift can be easily accounted for by estimating parameters such as F_ST_ and N_e_ (Wang et al., 2016). Only recently, with the advent of high-resolution genomes, the characterization of evolutionary adaptation at the genotypic level became a real possibility for many taxa. This is especially relevant for species that experience varying selective pressures across heterogeneous environments, leading to local adaptation.

Local adaptation refers to the increase in the frequency of a particular set of advantageous alleles under a specific selective regime, in such a way that disrupting these allele networks could impart negative consequences to the fitness of a population in a particular site (Savolainen et al., 2013). Disregarding adaptation to explain species distributions could cause sensible effects over direct conservation efforts. For example, Aguirre-Liguori et al. (2021)  showed that accounting for local adaptation when modeling suitable habitats in a scenario of rapid climate change is crucial for differentiating between changes in adaptive variants as a response to climate and that of neutral variants as a function of demographic history. Therefore, the predicted ecological niches could vary wildly in the landscape if information about potentially adaptive loci is not properly handled.

Several genomic approaches can be used to detect the genetic basis of adaptation, each differing in the type of data required, the statistical methods applied, and the genomic signatures they uncover (see Hoban et al., 2016; Ahrens et al., 2018; Rees et al., 2020; Wadgymar et al., 2022 for detailed information). For example, genetic differentiation outlier tests identify SNPs that show unusually high differentiation among populations compared to neutral expectations (commonly measured by the fixation index - F_ST_), suggesting selection at those loci. Genetic-environment association (GEA) analyses go further by correlating allele frequencies with environmental variables, thereby detecting loci potentially under spatially varying selection (Rellstab et al., 2015; Lasky et al., 2022). Genome-wide association studies (GWAS) associate genome-wide SNP variation with phenotypic or fitness traits across many individuals, thus allowing identification of genetic variants contributing to adaptation. Finally, population-specific selective sweep analyses detect genomic regions with extended haplotype homozygosity in one population, indicating recent strong positive selection (Rees *et al*., 2020). Together, these methods offer complementary opportunities to understand the genetic architecture underlying local adaptation, which can range from the influence of single genes with large effects to complex polygenic architectures involving many loci of small effects.

These approaches have revealed associations between genotypes, phenotypes and environmental variables; however, such signs should be interpreted as preliminary leads that require additional information (dominance and pleiotropy, epistasis, amount of linkage disequilibrium) and, ideally, functional validation to confirm their effects on fitness (Kardos et al., 2021). Moreover, the specific environmental factors driving local adaptation remain unknown in most cases, and researchers often assume that correlations with climatic variables correspond to agents of selection (Lasky et al., 2022; Wadgymar et al., 2022; Booker, 2024). A major remaining challenge is to demonstrate, through experimental analyses, the direct fitness effects and adaptive significance of the identified genomic regions. 

Local adaptation is particularly predicted for species with environmental gradients over their geographical distribution, large N_e_, and low migration rates (Funk et al., 2012). This pattern is exemplified by the Harbor porpoise, where salinity gradients across its range have led to subtle population structure, as revealed by GEA analyses based on genome-wide SNPs and the identification of candidate genes linked to salinity adaptation (Celemín et al., 2025). 

A typical management scenario in which local adaptation becomes of major concern is the guided ex situ mating, and translocation or reintroduction of (captive) individuals into the wild. In these scenarios, outbreeding depression disrupts co-adapted gene networks and locally selected phenotypes of ecological importance, such as in the case of the European alpine ibex, whose reintroduction caused population decline due to divergent locally adapted reproductive seasons (Leigh et al., 2021). Thus, screening for local adaptation should be incorporated into management decisions (Flanagan et al., 2018). This approach has been applied in the Eurasian lynx, where two distinct adaptive units were identified, differing in their local environmental conditions related to snow precipitation and daily temperature fluctuations (Bazzicalupo et al., 2023). It was therefore suggested that reintroduction or reinforcement efforts should account for these adaptive units when selecting source populations for translocation. Another example is the Pacific salmon, in which the genetic basis of variation in spawning timing was identified, where a single locus, GREB1L, controls the distinction between early and late spawning populations (Prince et al., 2017). However, the authors argue that both alleles underlying these different behaviors are critical for the persistence of the species, especially in the current climate change scenario, and therefore should be integrated into the species conservation plans.

In many cases, management programs aim to maintain - or even increase - genetic variability within and among populations. Some interesting cases involve introgression (i.e. the introduction of genetic material of one species into the genome of another through hybridization and back-crossing), which can eventually introduce adaptive alleles and may add essential genetic diversity that promotes adaptation in rapidly changing environments (Hedrick, 2013). For example, the development of resistance to herbivory was associated with introgressed alleles in *Helianthus* hybrids, and the ability to survive flooding was artificially demonstrated in introgressed individuals of the plants *Iris fulva* and *Iris brevicaulis* (Suarez-Gonzalez et al., 2018). In many taxa where interspecific hybridization is a common and natural event, adaptive introgression may play a significant role in the long-term adaptation of species and their capacity to withstand environmental changes.

Planning, monitoring, and re-evaluation are critical steps for a successful management plan that accounts for local adaptation. This includes being able to identify and define conservation units in terms of their genomic composition, demographic connectivity, evolutionary history, and geographical range (Barbosa et al., 2018). This approach is especially important when resources are limited and prioritizing populations for conservation is needed. Traditionally, evolutionarily significant units (ESUs) have been proposed to represent major breaks in genetic diversity between populations within species. With advances in genomics, Funk et al. (2012)  further refined this framework by identifying two categories within ESUs: Management Units (MUs), inferred from neutral variants and representing groups of populations that are demographically independent from each other; and Adaptive Units (AUs), which distinguish populations presenting (putatively) adaptive variants that confer advantages under specific environmental conditions. The implementation of adaptive unit strategies across multiple species (Barbosa *et al*., 2018; Miller et al., 2023; Hoste et al., 2024; Yang et al., 2025) has contributed to the preservation of genetic diversity that might otherwise be overlooked.

Although genomic-driven conservation strategies are promising, researchers caution that such approaches can be challenging due to the complex genetic basis underlying phenotypic traits and the risk of unintended consequences, such as the loss of genetic variation and evolutionary potential (Kardos and Shafer 2018). It is advisable to conduct a cost-benefit analysis to evaluate strategies for maintaining genome-wide genetic diversity against those targeting specific adaptive variants (Fernandez-Fournier et al., 2021). Indeed, prioritizing populations for conservation solely based on their genetic distinctiveness - whether neutral or adaptive - may not lead to optimal outcomes for the species (Weeks et al., 2016). 

While comparing genomic variability within and between populations of a species may provide valuable insight to guide management efforts, understanding the biological and ecological impact of adaptive variation - and the connection between genotypes and phenotypes - requires an interdisciplinary approach which enables a more appropriate conservation planning.

## Comparative genomics applied to biodiversity conservation

**Figure 3-:**
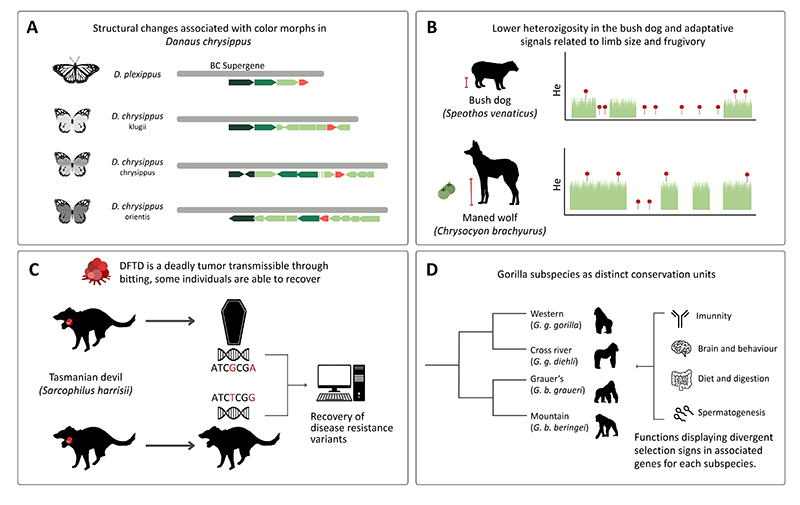
Examples of comparative genomics applied to biodiversity conservation. A) Structural rearrangements within BC supergene that are associated with three distinct color patterns in the African monarch butterfly (*Danaus chrysippus*). B) A comparative study of South American canids: the bush dog and its closest relative, the maned wolf, present different selection signals related to limb size. In addition, the bush dog has the lowest heterozygosity within the family, and the maned wolf present also metabolic adaptations related to frugivory. C) Tasmanian devils are affected by the Devil Facial Tumor Disease (DFTD), a rare transmissible and deadly tumor. Tasmanian devils recovered from DTFD have signals of disease resistance variants. D) A comprehensive study of the four subspecies of gorillas revealed different signs of local adaptation for distinctive sets of genes in the same bodily functions.

Comparative genomics is a broad research field focused on analyzing and contrasting the genomes of different organisms to identify both their conserved features and variations (Hardison, 2003). However, functionally annotated genomes are required to undertake detailed comparisons, that is, genomes with information about where genes, regulatory elements, and other features are located, and their predicted functions. Thus, a sequenced, assembled and, particularly, annotated genome is a considerable limiting factor in meaningful comparative genomics studies (Housman and Gilad 2020). The extensive global, regional, and local initiatives aimed at producing annotated reference genomes hold great promise. The availability of reference genomes for hundreds of taxa may transform comparative genomics into a powerful tool for addressing questions directly relevant to biodiversity conservation (Figure 3), thereby changing a field that has historically focused on other aspects of biological evidence (Grueber, 2015).

Comparative genomic studies are also used to link genotypes and phenotypes, uncovering molecular mechanisms underlying specific traits with significant implications for biodiversity conservation, biotechnology, health, and evolutionary research. For example, comparative genomics has provided key insights into the molecular foundations of complex traits like longevity, cancer, virus susceptibility and resistance, vocal learning, multicellularity, and adaptations to extreme environments (reviewed in Hilgers and Hiller, 2025; Pereira Lobo et al., 2025). A particular case in conservation comes from studies on the endangered Tasmanian devil, where comparative and population genomics have been used to identify genetic variants associated with resistance to the transmissible devil facial tumor disease (Figure 3C), guiding breeding programs aimed at enhancing disease resilience (Hohenlohe et al., 2019).

Contrasting the genomic content of threatened and non-threatened species can be used to better understand the evolutionary background of the former; it can help to identify individuals with low genetic diversity as well as individuals carrying possible adaptive variants, both features worth considering while managing species and delimiting ESUs (Funk et al., 2012; Grueber, 2015). For example, a comparative study of South American canids identified the bush dog as having the lowest genome-wide heterozygosity and the highest proportion of harmful variants, highlighting a pressing conservation concern (Chavez et al., 2022). They also identified signatures of positive selection in genes likely underlying the disparate limb proportions in the bush dog and the maned wolf, and metabolic genes associated with frugivory in the maned wolf (Figure 3B). Similarly, a comparative genomics study of the four subspecies of gorillas showed that eastern gorillas are less genetically diverse than the western gorillas, but they also present a smaller genetic load, probably the result of genetic purging after drastic population reductions in the past (van der Valk et al., 2024). Moreover, this study showed that each subspecies had indicators of local adaptation, supporting their recognition as distinct evolutionary units (Figure 3D).

Other broader comparative studies have revealed some key determinants of genetic diversity. For example, the comparative transcriptome analyses of 76 animal species found that “ecological strategy” plays a major role in shaping genetic diversity (Romiguier et al., 2014). Specifically, they showed that long-lived or low-fecundity species with brooding behaviors tend to exhibit lower genetic diversity than short-lived or highly fecund species. These findings suggest that conservation decisions should consider life-history traits in addition to raw measures of genetic diversity.

The growing availability of genomic data has also provided increasing evidence that (natural) introgressive hybridization plays an important role in facilitating species adaptation (i.e., adaptive introgression). For instance, genomic analysis of wolves and coyotes revealed that adaptive introgression has facilitated the expansion of coyotes into new habitats while maintaining distinct genetic lineages (vonHoldt et al., 2016). It illustrates how hybridization can, when not created and/or exacerbated by human activity, have beneficial consequences for conservation in particular cases and taxa. Overall, the examples displayed here convey how comparative genomics helps conservation endeavors by linking genomic variation to adaptive potential, guiding management decisions that are in line with evolutionary processes.

Evaluating genomic synteny between species provides a powerful framework to investigate genome structure and how gene function has evolved over time. By aligning and contrasting genomic regions across taxa, researchers can identify conserved and rearranged segments that reflect both shared ancestry and lineage-specific adaptations. Several studies have shown highly conserved synteny within major groups, such as felids (Bredemeyer et al., 2023), sea turtles (Arantes et al., 2025a) and birds (Zhang et al., 2014; Granger-Neto et al., 2025), suggesting that large-scale genomic architecture can remain stable over millions of years of evolution. On the other hand, comparative analyses have also revealed that chromosomal rearrangements play a key role in facilitating local adaptation and phenotypic diversification (Schneller et al., 2025). For example, rearrangements encompassing the BC supergene (Figure 3A) are associated with three distinct wing color morphs in the African monarch butterfly (Kim et al., 2022), highlighting how structural genomic variation can generate adaptive diversity. Thus, exploring patterns of conserved and rearranged synteny across species provides critical insights into the balance between genome stability and evolutionary innovation that underlies adaptive evolution.

Comparative genomics can also provide us with relevant data to characterize conservation units more precisely, considering aspects like the consequences of hybridization - whether natural or human-driven - along with identifying signatures of threats to the conservation status of a species (Grueber 2015). For example, genome-wide analyses can help detecting locally adapted populations that should be treated as distinct management units, reveal whether hybridization introduces maladaptive or beneficial alleles, identify selection pressures driving ecological or physiological divergence, and pinpoint immune or stress-response genes linked to survival under environmental change (Campagna and Toews, 2022; Shao et al., 2023). Collectively, these approaches allow for conservation strategies that are informed not only by demography and geography, but also by the evolutionary and functional context of populations.

With the ongoing emergence of reference-level genomes for major taxonomic groups, the new challenge is to functionally annotate the genome and infer patterns of gene evolution, which requires understanding orthologous relationships (Langschied et al., 2024). Genes can be predicted with the help of transcriptomic data or inferred with information from related organisms. Since obtaining RNAseq data for many endangered species is challenging (because it requires fresh, well-preserved samples), projecting gene predictions and functional annotations from closely related species has been proposed and applied as a strategy to generate annotations across the eukaryotic species (Guigó, 2023). Moving forward, the field of comparative genomics will need to develop specialized approaches for identifying gene orthologs of short proteins and noncoding RNAs, functionally characterizing novel gene families, and integrating machine-learning-predicted RNA and protein structures with orthology-based analyses, facilitating a more comprehensive understanding of genome function and evolution (Langschied *et al*., 2024).

## Environmental DNA analyses for biodiversity monitoring

Global biodiversity indicators show steep declines that are expected to accelerate without coordinated conservation measures (Jenkins et al., 2013). This highlights the need to improve knowledge of species distributions and community composition through innovative sampling approaches capable of accurately assessing current biodiversity. The advent of environmental DNA (eDNA) represents a promising revolution in biodiversity monitoring (Bohmann et al., 2014). Organisms continuously release genetic material into the environment, either within cells or as free extracellular DNA and RNA, through animal tissues, hair, feces, urine, mucus, and saliva, as well as plant trunks, leaves, pollen, seeds, and fruits (Barnes and Turner, 2016; Çevik and Çevik, 2025). This genetic material can be non-invasively recovered from soil, water, air, or other environmental samples, enabling the detection of species without the need for direct observation or physical capture of individuals (Thomsen and Willerslev, 2015; Cristescu and Hebert, 2018).

Environmental DNA has proven invaluable for species- and community-scale monitoring, as eDNA samples can simultaneously detect multiple target species or even capture community-wide biodiversity. It offers a promising approach to assess unknown geographic distribution and temporal dynamics of species (Thomsen and Willerslev, 2015; Kjær et al., 2022; Carraro et al., 2023; Perry et al., 2024), including cryptic, threatened, rare and invasive species (e.g., Sasso et al., 2017; Sales et al., 2020; Andrade et al., 2021). Recently, eDNA has also emerged as a non-invasive and low-cost approach for assessing population genetic features, detecting haplotypes, SNPs, and microsatellites to estimate genetic diversity and connectivity among populations, which is particularly valuable for conserving rare or poorly known species (Andres et al., 2023; Couton et al., 2023).

At the community level, eDNA enables a comprehensive characterization of biodiversity, providing insights into species richness, relative abundances, community connectivity, and temporal changes in communities (Thomsen and Willerslev 2015; Cristescu and Hebert 2018). Because eDNA can persist in soils and sediments for long periods, especially in frozen environments such as permafrost, it allows the reconstruction of ancient communities and the investigation of historical changes in community composition, extinctions, biological invasions, and their potential drivers (Thomsen and Willerslev, 2015; Ellegaard et al., 2020). In contexts where rapid ecological shifts require near-real-time monitoring and assessment, such as climate change, environmental accidents, or other human activities (e.g., factories, power plants, and other industrial developments), eDNA also represents a highly responsive approach (Thomsen and Willerslev, 2015; Berry et al., 2019; DiBattista et al., 2022). For instance, eDNA has been applied to monitor marine vertebrates following the Fundão/Mariana tailings dam failure in Brazil (Lines et al., 2023), to evaluate plant communities near mining areas (Martins et al., 2024), and to assess fish communities in the Itaipu reservoir (Dal Pont et al., 2021), most of them yielding better results than conventional sampling methods (Iacaruso et al., 2025).

In addition to being dispersed into the environment, DNA can move across trophic levels, transferring from prey to predator or from host to parasite. Approaches using DNA extracted from gut, stomach, or fecal contents - collectively known as dietary DNA (dDNA) (De Sousa et al., 2019) - allow simultaneous assessment of dietary habits and biodiversity. Scat DNA (sDNA) recovered from the feces of mammals, birds, reptiles, and fishes (e.g., Quéméré et al., 2021; Kurita and Toda, 2022; Bookwalter et al., 2023; Sato, 2024; Vasiliadis et al., 2024) provides higher taxonomic resolution than visual identification of digested prey, often reaching species level even for small or soft-bodied organisms, and revealing previously undocumented trophic interactions (De Sousa *et al*., 2019; Ando *et al*., 2020). Biodiversity and species’ dietary habits can also be inferred from invertebrate-derived DNA (iDNA) recovered from the gut contents of hematophagous, saprophagous and coprophagous invertebrates (e.g., Fahmy et al., 2023; Fernandes et al., 2023; Lee et al., 2023; Saranholi et al., 2023; Massey et al., 2025). iDNA has been primarily used to assess vertebrate biodiversity, but it can also serve as a powerful forensic tool for identifying trafficked wildlife (Williams et al., 2020). By integrating information on trophic interactions and community composition, dDNA contributes substantially to ecological research and directly supports conservation efforts, including the detection and monitoring of elusive or endangered species that are often missed by conventional survey methods (Yuan et al., 2025).

Some eDNA studies have focused on particular species of interest, such as rare, threatened, or invasive taxa, using species-specific markers combined with techniques such as quantitative PCR (qPCR), digital PCR (dPCR), or CRISPR-Cas (e.g., Thomsen et al., 2012; Gargan et al., 2017; Andrade et al., 2021; Williams et al., 2021; Smith et al., 2022). Alternatively, universal primer sets that typically amplify short DNA fragments of standard genetic markers can be combined with high-throughput sequencing to simultaneously detect and identify multiple species or even entire communities, an approach known as metabarcoding (Creer et al., 2016; Ruppert et al., 2019; Wang et al., 2023). A major limitation of eDNA metabarcoding is the incomplete and uneven taxonomic coverage of reference sequences - among the approximately 2 million described eukaryotic species (Lewin et al., 2018), only 26% are represented in the NCBI Genbank (Sayers et al., 2022) - as well as the occurrence of taxonomic errors, misidentifications, and outdated names (Keck et al., 2023). Moreover, eDNA metabarcoding relies on PCR amplification, which can introduce biases such as primer affinity differences, preferential amplification of dominant species, underrepresentation of rare species, chimeric or artifactual sequences, amplification errors, and susceptibility to contamination (Cristescu and Hebert 2018). Nevertheless, initiatives to reduce these challenges, such as the design and adaptation of primers to specific taxonomic groups, have been proposed (e.g., Chaves et al., 2025).

To overcome limitations of metabarcoding, eDNA metagenomics represents the next frontier in biodiversity monitoring, enabling a more comprehensive and accurate depiction of ecosystem diversity through the direct sequencing of all DNA fragments in environmental samples via shotgun sequencing. This approach enables simultaneous taxonomic profiling across all domains of life, functional gene annotation, assessment of entire mitogenomes, metabolic pathway inference, and the reconstruction of Metagenome-Assembled Genomes (MAGs) (Quince et al., 2017; Zhou et al., 2022; Hauptfeld et al., 2024). Integrated with metatranscriptomic, metaproteomic, and metabolomic datasets, it provides a multidimensional understanding of ecosystem structure and function (Aguiar-Pulido et al., 2016; Cordier et al., 2021). This approach can be applied to reveal the diversity of Bacteria, Archaea, and Eukaryota domains, including complex marine faunal assemblages (Cowart et al., 2018; Manu and Umapathy, 2023).

However, eDNA metagenomics faces some important challenges, including the need for high-quality DNA, deep sequencing coverage, considerable financial and computational resources, and particularly, the lack of reference genomes for most taxa (Quince et al., 2017; Blackman et al., 2023; Curto et al., 2025; Patin et al., 2025). Remarkably, as of 2025, reference genomes (roughly 20,000) are available for less than 1% of all eukaryotic species in the NCBI Genomes database.

Environmental RNA (eRNA) has recently emerged as a complementary tool to eDNA for biodiversity monitoring, providing information not only about species presence but also about biological activity (Cristescu, 2019; Marshall et al., 2021; Veilleux et al., 2021). However, eRNA is highly unstable and has a short persistence in the environment, providing a more instantaneous snapshot of the actively living community and improving the detection of rare or low-abundance taxa (Cristescu 2019; Wood et al., 2020; Marshall *et al*., 2021; Veilleux *et al*., 2021). In addition, since RNA expression varies with developmental stage, sex, and physiological condition, eRNA-based analyses can reveal functional and phenotypic information that eDNA cannot capture (Yates et al., 2021).

**Figure 4-:**
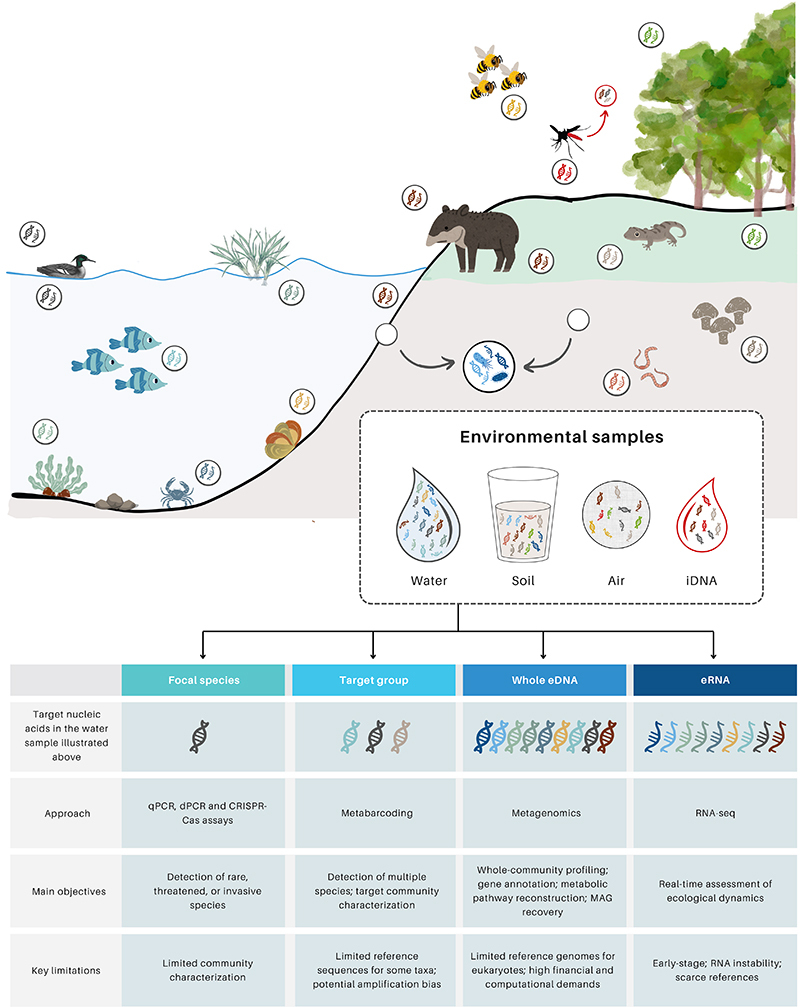
Conceptual representation of environmental nucleic acid approaches. Environmental DNA (eDNA) and RNA (eRNA) can be obtained from various substrates such as water, soil, air, and biological residues (iDNA). Depending on the target nucleic acids and analytical methods, studies can focus on individual species, particular taxonomic groups, or entire communities, as depicted in the summarizing table, focused on the water sample as an example.

Environmental DNA and RNA monitoring represent a significant advance in our molecular understanding of biodiversity, revolutionizing how we reconstruct past and present distributions of native and invasive species, assess changes in community composition, and unravel ecological interactions, central questions in conservation biology (Figure 4). Far beyond producing simple species inventories, these approaches can be integrated with other molecular (some of which are discussed in other sections of this review), ecological datasets (e.g., ecological niche modeling, Brantschen et al., 2024), and other innovative technologies (e.g., remote sensing, Randin et al., 2020) to enable more comprehensive, predictive, and effective conservation strategies (Nielsen et al., 2023; Bernatchez et al., 2024). Such integrative frameworks are essential to guide biodiversity conservation in a world undergoing rapid and profound environmental change. Besides, a recent groundbreaking study with ancient environmental DNA analyses of soil revealed the biodiversity of a 2-million-year-old (Pleistocene) ecosystem in Greenland (Kjær et al., 2022). The possibility of reconstructing ancient biological communities using eDNA metagenomics opens exciting new perspectives to understand biodiversity dynamics in the deep past, and to envisage more appropriate conservation strategies in the long term.

## Future perspectives

The proliferation of genomic data has brought about the challenge of big data management and analysis. Advances in bioinformatics have been critical to handling large genomic datasets, but increasingly, machine learning and artificial intelligence (AI) are being leveraged to improve interpretation. AI algorithms can identify patterns in genetic variation that correlate with ecological variables, predict adaptive responses to climate change, and model species distributions under future scenarios (van Oosterhout, 2024). 

Many new developments are envisaged with the increasing availability of high-resolution genomes and new analytical methods joining bioinformatics and artificial intelligence. It is a brand-new scientific achievement, which demands a careful and thoughtful reflection about what we mean by biodiversity conservation and the potential unexpected effects. For example, the use of genome editing and cloning has been claimed by some scientists (van Oosterhout et al., 2025) as a possible alternative for species rescue. However, the authors with competing interests (declared in the article) ignore that pre-Anthropocene extinction is part of the evolutionary history, and their extremely expensive “rescue” can also be an anthropic impact on natural habitats worldwide that could be preserved with a more limited funding.

The complete genomes with phased chromosomes of many taxa will increase the power of comparative methods, and when coupled with functional validation, it can be used to characterize many genotype-phenotype associations, perhaps the most important scientific gap we have in biology today. It is essential to understand the adaptation of populations to different selective pressures in the landscape and through time. Besides, informative variant datasets can now be generated from aligned collections of high-quality genomes and displayed as pangenome graphs to adequately represent genetic diversity within a population or species (Secomandi et al., 2025).

Finally, genomic information needs careful translation to practical conservation management (Holderegger et al., 2019). A long‐term engagement between academics and conservation practitioners is also needed, as observed in some Brazilian Action Plans of Conservation (PAN) composed by members of academic, government and social organizations (ICMBio, 2024).

## Concluding remarks

Genomic technologies are conducting a new era of wildlife conservation, offering powerful tools to understand and monitor biodiversity, manage endangered species, and anticipate ecological challenges. Recent studies have demonstrated genomics to increase the accuracy of conservation decision-making applied to many taxa and ecoregions worldwide. While many ethical, economic, logistical, and policy challenges remain, the integration of genomics data with other scientifically relevant knowledge about species and ecosystems promises transformative impacts on conservation practice.

## Data Availability

All data used in this manuscript are available in the cited references.

## References

[B1] Aguiar-Pulido V, Huang W, Suarez-Ulloa V, Cickovski T, Mathee K, Narasimhan G (2016). Metagenomics, metatranscriptomics, and metabolomics approaches
for microbiome analysis: Supplementary issue: Bioinformatics methods and
applications for big metagenomics data. Evol Bioinform.

[B2] Aguirre-Liguori JA, Ramírez-Barahona S, Gaut BS (2021). The evolutionary genomics of species’ responses to climate
change. Nat Ecol Evol.

[B3] Ahrens CW, Rymer PD, Stow A, Bragg J, Dillon S, Umbers KDL, Dudaniec RY (2018). The search for loci under selection: trends, biases and
progress. Mol Ecol.

[B4] Allendorf FW, Hohenlohe PA, Luikart G (2010). Genomics and the future of conservation genetics. Nat Rev Genet.

[B5] Ando H, Mukai H, Komura T, Dewi T, Ando M, Isagi Y (2020). Methodological trends and perspectives of animal dietary studies
by noninvasive fecal DNA metabarcoding. Environ DNA.

[B6] Andrade PDBD, Razzolini E, Baggio RA (2021). I See Golden Mussel! They are everywhere! Environmental DNA
supports widespread dissemination of Limnoperna fortunei in hydrographic
basins in the Paraná State, Brazil. Braz Arch Biol Technol.

[B7] Andres KJ, Lodge DM, Sethi SA, Andrés J (2023). Detecting and analysing intraspecific genetic variation with
eDNA: From population genetics to species abundance. Mol Ecol.

[B8] Andrews KR, Good JM, Miller MR, Luikart G, Hohenlohe PA (2016). Harnessing the power of RADseq for ecological and evolutionary
genomics. Nat Rev Genet.

[B9] Arantes LS, Brown T, De Panis D, Whiting SD, Young EJ, LaCasella EL, Carvajal GA, Kennedy A, Edmunds D, Bentley BP (2025). Haplotype-resolved reference genomes of the sea turtle clade
unveil ultra-syntenic genomes with hotspots of divergence. GigaScience.

[B10] Arantes LS, De Panis D, Miranda FR, Santos FR, Hiller M, Mazzoni CJ (2025). Genomic signatures in maned three-toed sloths from ancient to
recent environmental changes in Brazil’s threatened Atlantic
Forest. Mol Ecol.

[B11] Barbosa S, Mestre F, White TA, Paupério J, Alves PC, Searle JB (2018). Integrative approaches to guide conservation decisions: Using
genomics to define conservation units and functional
corridors. Mol Ecol.

[B12] Barnes MA, Turner CR (2016). The ecology of environmental DNA and implications for
conservation genetics. Conserv Genet.

[B13] Bazzicalupo E, Ratkiewicz M, Seryodkin IV, Okhlopkov I, Galsandorj N, Yarovenko YA, Ozolins J, Saveljev AP, Melovski D, Gavashelishvili A (2023). Genome-environment association analyses reveal geographically
restricted adaptive divergence across the range of the widespread Eurasian
carnivore Lynx lynx (Linnaeus, 1758). Evol Appl.

[B14] Bernatchez L, Ferchaud A-L, Berger CS, Venney CJ, Xuereb A (2024). Genomics for monitoring and understanding species responses to
global climate change. Nat Rev Genet.

[B15] Berry TE, Saunders BJ, Coghlan ML, Stat M, Jarman S, Richardson AJ, Davies CH, Berry O, Harvey ES, Bunce M (2019). Marine environmental DNA biomonitoring reveals seasonal patterns
in biodiversity and identifies ecosystem responses to anomalous climatic
events. PloS Genet.

[B16] Bertorelle G, Raffini F, Bosse M, Bortoluzzi C, Iannucci A, Trucchi E, Morales HE, van Oosterhout C (2022). Genetic load: Genomic estimates and applications in non-model
animals. Nat Rev Genet.

[B17] Blackman RC, Walser J, Rüber L, Brantschen J, Villalba S, Brodersen J, Seehausen O, Altermatt F (2023). General principles for assignments of communities from eDNA: Open
versus closed taxonomic databases. Environ DNA.

[B18] Bohmann K, Evans A, Gilbert MTP, Carvalho GR, Creer S, Knapp M, Yu DW, De Bruyn M (2014). Environmental DNA for wildlife biology and biodiversity
monitoring. Trends Ecol Evol.

[B19] Booker TR (2024). The structure of the environment influences the patterns and
genetics of local adaptation. Evol Lett.

[B20] Bookwalter J, Niyas AMM, Caballero-López B, Villari C, Claramunt-López B (2023). Fecal matters: implementing classical Coleoptera species lists
with metabarcoding data from passerine bird feces. J Insect Conserv.

[B21] Bosse M, van Loon S (2022). Challenges in quantifying genome erosion for
conservation. Front Genet.

[B22] Bowden R, MacFie TS, Myers S, Hellenthal G, Nerrienet E, Bontrop RE, Freeman C, Donnelly P, Mundy NI (2012). Genomic tools for evolution and conservation in the Chimpanzee:
Pan troglodytes ellioti is a genetically distinct population. PLoS Genet.

[B23] Brandies P, Peel E, Hogg CJ, Belov K (2019). The value of reference genomes in the conservation of threatened
species. Genes.

[B24] Brantschen J, Fopp F, Adde A, Keck F, Guisan A, Pellissier L, Altermatt F (2024). Habitat suitability models reveal the spatial signal of
environmental DNA in riverine networks. Ecography.

[B25] Bredemeyer KR, Hillier L, Harris AJ, Hughes GM, Foley NM, Lawless C, Carroll RA, Storer JM, Batzer MA, Rice ES (2023). Single-haplotype comparative genomics provides insights into
lineage-specific structural variation during cat evolution. Nat Genet.

[B26] Campagna L, Toews DPL (2022). The genomics of adaptation in birds. Curr Biol.

[B27] Campos DP, Granger- HP, J ES, Faux P, Santos FR (2023). Population genomics of the critically endangered Brazilian
merganser. Animals.

[B28] Campos DP, Granger- HP, J ES, Buzatti RS, Santos FR (2024). Genetic monitoring of the captive population of the critically
endangered brazilian merganser (Mergus octosetaceus). Birds.

[B29] Carraro L, Blackman RC, Altermatt F (2023). Modelling environmental DNA transport in rivers reveals highly
resolved spatio-temporal biodiversity patterns. Sci Rep.

[B30] Celemín E, Autenrieth M, Roos A, Pawliczka I, Quintela M, Lindstrøm U, Benke H, Siebert U, Lockyer C, Berggren P (2025). Evolutionary history and seascape genomics of Harbour porpoises
(Phocoena phocoena) across environmental gradients in the North Atlantic and
adjacent waters. Mol Ecol Resour.

[B31] Çevik T, Çevik N (2025). Environmental DNA (eDNA): A review of ecosystem biodiversity
detection and applications. Biodivers Conserv.

[B32] Charlesworth D, Willis JH (2009). The genetics of inbreeding depression. Nat Rev Genet.

[B33] Chaves BRN, Santos- JE, Santos FR (2025). Metabarcoding markers and a reference database for neotropical
mammals. Conserv Genet Resour.

[B34] Chavez DE, Gronau I, Hains T, Dikow RB, Frandsen PB, Figueiró HV, Garcez FS, Tchaicka L, de Paula RC, Rodrigues FHG (2022). Comparative genomics uncovers the evolutionary history,
demography, and molecular adaptations of South American
canids. Proc Natl Acad Sci U S A.

[B35] Christiansen H, Heindler FM, Hellemans B, Jossart Q, Pasotti F, Robert H, Verheye M, Danis B, Kochzius M, Leliaert F (2021). Facilitating population genomics of non-model organisms through
optimized experimental design for reduced representation
sequencing. BMC Genomics.

[B36] Cordier T, Alonso-Sáez L, Apothéloz-Perret-Gentil L, Aylagas E, Bohan DA, Bouchez A, Chariton A, Creer S, Frühe L, Keck F (2021). Ecosystems monitoring powered by environmental genomics: A review
of current strategies with an implementation roadmap. Mol Ecol.

[B37] Couton M, Viard F, Altermatt F (2023). Opportunities and inherent limits of using environmental DNA for
population genetics. Environ DNA.

[B38] Cowart DA, Murphy KR, Cheng C-HC (2018). Metagenomic sequencing of environmental DNA reveals marine faunal
assemblages from the West Antarctic Peninsula. Mar Genomics.

[B39] Creer S, Deiner K, Frey S, Porazinska D, Taberlet P, Thomas WK, Potter C, Bik HM (2016). The ecologist’s field guide to sequence‐based identification of
biodiversity. Methods Ecol Evol.

[B40] Cristescu ME (2019). Can environmental RNA revolutionize biodiversity
science?. Trends Ecol Evol.

[B41] Cristescu ME, Hebert PDN (2018). Uses and misuses of environmental DNA in biodiversity science and
conservation. Annu Rev Ecol Evol Syst.

[B42] Curik I, Ferenčaković M, Sölkner J (2014). Inbreeding and runs of homozygosity: A possible solution to an
old problem. Livest Sci.

[B43] Curto M, Veríssimo A, Riccioni G, Santos CD, Ribeiro F, Jentoft S, Alves MJ, Gante HF (2025). Improving whole biodiversity monitoring and discovery with
environmental DNA metagenomics. Mol Ecol Resour.

[B44] Dal Pont G, Duarte Ritter C, Agostinis AO, Stica PV, Horodesky A, Cozer N, Balsanelli E, M OSM, Henn C, Ostrensky A (2021). Monitoring fish communities through environmental DNA
metabarcoding in the fish pass system of the second largest hydropower plant
in the world. Sci Rep.

[B45] De Sousa LL, Silva SM, Xavier R (2019). DNA metabarcoding in diet studies: Unveiling ecological aspects
in aquatic and terrestrial ecosystems. Environ DNA.

[B46] DeWoody JA, Harder AM, Mathur S, Willoughby JR (2021). The long-standing significance of genetic diversity in
conservation. Mol Ecol.

[B47] DiBattista JD, Fowler AM, Riley IJ, Reader S, Hay A, Parkinson K, Hobbs J-PA (2022). The use of environmental DNA to monitor impacted coastal
estuaries. Mar Pollut Bull.

[B48] Díez-del-Molino D, Sánchez-Barreiro F, Barnes I, Gilbert MTP, Dalén L (2018). Quantifying temporal genomic erosion in endangered
species. Trends Ecol Evol.

[B49] Dobzhansky T (1957). Mendelian populations as genetic systems. Cold Spring Harb Symp Quant Biol.

[B50] Ebenezer TE, Muigai AWT, Nouala S, Badaoui B, Blaxter M, Buddie AG, Jarvis ED, Korlach J, Kuja JO, Lewin HA (2022). Africa: Sequence 100,000 species to safeguard
biodiversity. Nature.

[B51] Edmands S (2007). Between a rock and a hard place: Evaluating the relative risks of
inbreeding and outbreeding for conservation and management. Mol Ecol.

[B52] Ellegaard M, Clokie MRJ, Czypionka T, Frisch D, Godhe A, Kremp A, Letarov A, McGenity TJ, Ribeiro S, John Anderson N (2020). Dead or alive: Sediment DNA archives as tools for tracking
aquatic evolution and adaptation. Commun Biol.

[B53] Fahmy M, Andrianoely D, Wright PC, Hekkala E (2023). Leech‐derived iDNA complements traditional surveying methods,
enhancing species detections for rapid biodiversity sampling in the
tropics. Environ DNA.

[B54] Feng S, Fang Q, Barnett R, Li C, Han S, Kuhlwilm M, Zhou L, Pan H, Deng Y, Chen G (2019). The genomic footprints of the fall and recovery of the crested
Ibis. Curr Biol.

[B55] Fernandes K, Bateman PW, Saunders BJ, Bunce M, Bohmann K, Nevill P (2023). Use of carrion fly iDNA metabarcoding to monitor invasive and
native mammals. Conserv Biol.

[B56] Fernandez-Fournier P, Lewthwaite JMM, Mooers AØ (2021). Do we need to identify adaptive genetic variation when
prioritizing populations for conservation?. Conserv Genet.

[B57] Flanagan SP, Forester BR, Latch EK, Aitken SN, Hoban S (2018). Guidelines for planning genomic assessment and monitoring of
locally adaptive variation to inform species conservation. Evol Appl.

[B58] Formenti G, Theissinger K, Fernandes C, Bista I, Bombarely A, Bleidorn C, Ciofi C, Crottini A, Godoy JA, Höglund J (2022). The era of reference genomes in conservation
genomics. Trends Ecol Evol.

[B59] Fuentes-Pardo AP, Ruzzante DE (2017). Whole-genome sequencing approaches for conservation biology:
Advantages, limitations and practical recommendations. Mol Ecol.

[B60] Funk WC, McKay JK, Hohenlohe PA, Allendorf FW (2012). Harnessing genomics for delineating conservation
units. Trends Ecol Evol.

[B61] Gargan LM, Morato T, Pham CK, Finarelli JA, Carlsson JEL, Carlsson J (2017). Development of a sensitive detection method to survey pelagic
biodiversity using eDNA and quantitative PCR: A case study of devil ray at
seamounts. Mar Biol.

[B62] Granger- HP, Borges IRF, Cavalcanti DB, Campos DP, Horan K, Balacco J, O’Toole B, Tilley T, Jain N, Abueg L (2025). Chromosome-level genome assembly of the Brazilian merganser
(Mergus octosetaceus), a rare and elusive waterfowl species. bioRxiv.

[B63] Grueber CE (2015). Comparative genomics for biodiversity
conservation. Comput Struct Biotechnol J.

[B64] Guigó R (2023). Genome annotation: From human genetics to biodiversity
genomics. Cell Genomics.

[B65] Hardison RC (2003). Comparative genomics. PLoS Biol.

[B66] Hauptfeld E, Pappas N, Van Iwaarden S, Snoek BL, Aldas-Vargas A, Dutilh BE, Von Meijenfeldt FAB (2024). Integrating taxonomic signals from MAGs and contigs improves read
annotation and taxonomic profiling of metagenomes. Nat Commun.

[B67] Hedrick PW (2013). Adaptive introgression in animals: examples and comparison to new
mutation and standing variation as sources of adaptive
variation. Mol Ecol.

[B68] Hendricks S, Anderson EC, Antao T, Bernatchez L Forester BR, Garner B, Hand BK, Hohenlohe PA, Kardos M, Koop B (2018). Recent advances in conservation and population genomics data
analysis. Evol Appl.

[B69] Hilgers L, Hiller M (2025). Linking phenotype to genotype using comprehensive genomic
comparisons. Curr Opin Genet Dev.

[B70] Hoban S, Kelley JL, Lotterhos KE, Antolin MF, Bradburd G, Lowry DB, Poss ML, Reed LK, Storfer A, Whitlock MC (2016). Finding the genomic basis of local adaptation: pitfalls,
practical solutions, and future directions. Am Nat.

[B71] Hoban S, Archer FI, Bertola LD, Bragg JG, Breed MF, Bruford MW, Coleman MA, Ekblom R, Funk WC, Grueber CE (2022). Global genetic diversity status and trends: Towards a suite of
Essential Biodiversity Variables (EBVs) for genetic
composition. Biol Rev.

[B72] Hohenlohe PA, Funk WC, Rajora OP (2021). Population genomics for wildlife conservation and
management. Mol Ecol.

[B73] Hohenlohe PA, McCallum HI, Jones ME, Lawrance MF, Hamede RK, Storfer A (2019). Conserving adaptive potential: Lessons from Tasmanian devils and
their transmissible cancer. Conserv Genet.

[B74] Holderegger R, Balkenhol N, Bolliger J, Engler JO, Gugerli F, Hochkirch A, Nowak C, Segelbacher G, Widmer A, Zachos FE (2019). Conservation genetics: Linking science with
practice. Mol Ecol.

[B75] Hoste A, Capblancq T, Broquet T, Denoyelle L, Perrier C, Buzan E, Šprem N, Corlatti L, Crestanello B, Hauffe HC (2024). Projection of current and future distribution of adaptive genetic
units in an alpine ungulate. Heredity.

[B76] Housman G, Gilad Y (2020). Prime time for primate functional genomics. Curr Opin Genet Dev.

[B77] Humble E, Stoffel MA, Dicks K, Ball AD, Gooley RM, Chuven J, Pusey R, Remeithi MA, Koepfli K-P, Pukazhenthi B (2023). Conservation management strategy impacts inbreeding and mutation
load in scimitar-horned oryx. Proc Natl Acad Sci U S A.

[B78] Iacaruso NJ, Reves OP, Merkelz SJ, Waldrep CL, Davis MA (2025). A systematic review evaluating the performance of eDNA methods
relative to conventional methods for biodiversity monitoring. Ecography.

[B79] Chico Mendes Institute for Biodiversity Conservation (2024). Planning and Management of national action plans for threatened species
conservation: The Brazilian approach.

[B80] Jenkins CN, Pimm SL, Joppa LN (2013). Global patterns of terrestrial vertebrate diversity and
conservation. Proc Natl Acad Sci U S A.

[B81] Jeon JY, Black AN, Heenkenda EJ, Mularo AJ, Lamka GF, Janjua S, Brüniche-Olsen A, Bickham JW, Willoughby JR, DeWoody JA (2024). Genomic diversity as a key conservation criterion:
Proof-of-concept from mammalian whole-genome resequencing
data. Evol Appl.

[B82] Kardos M, Shafer ABA (2018). The Peril of gene-targeted conservation. Trends Ecol Evol.

[B83] Kardos M, Taylor HR, Ellegren H, Luikart G, Allendorf FW (2016). Genomics advances the study of inbreeding depression in the
wild. Evol Appl.

[B84] Kardos M, Åkesson M, Fountain T, Flagstad Ø, Liberg O, Olason P, Sand H, Wabakken P, Wikenros C, Ellegren H (2018). Genomic consequences of intensive inbreeding in an isolatedwolf
population. Nat Ecol Evol.

[B85] Kardos M, Armstrong EE, Fitzpatrick SW, Hauser S, Hedrick PW, Miller JM, Tallmon DA, Funk WC (2021). The crucial role of genome-wide genetic variation in
conservation. Proc Natl Acad Sci U S A.

[B86] Kardos M, Zhang Y, Parsons KM, A Y, Kang H, Xu X, Liu X, Matkin CO, Zhang P, Ward EJ (2023). Inbreeding depression explains killer whale population
dynamics. Nat Ecol Evol.

[B87] Keck F, Couton M, Altermatt F (2023). Navigating the seven challenges of taxonomic reference databases
in metabarcoding analyses. Mol Ecol Resour.

[B88] Khan A, Patel K, Shukla H, Viswanathan A, van der Valk T, Borthakur U, Nigam P, Zachariah A, Jhala YV, Kardos M (2021). Genomic evidence for inbreeding depression and purging of
deleterious genetic variation in Indian tigers. Proc Natl Acad Sci U S A.

[B89] Kim K-W, De-Kayne R, Gordon IJ, Omufwoko KS, Martins DJ, ffrench-Constant R, Martin SH (2022). Stepwise evolution of a butterfly supergene via duplication and
inversion. Philos Trans R Soc B Biol Sci.

[B90] Kjær KH, Winther Pedersen M, De Sanctis B, De Cahsan B, Korneliussen TS, Michelsen CS, Sand KK, Jelavić S, Ruter AH, Schmidt AMA (2022). A 2-million-year-old ecosystem in Greenland uncovered by
environmental DNA. Nature.

[B91] Kurita T, Toda M (2022). Comparison of morphological identification and DNA metabarcoding
for dietary analysis of faeces from a subtropical lizard. Wildl Res.

[B92] Kyriazis CC, Robinson JA, Lohmueller KE (2025). Long runs of homozygosity are reliable genomic markers of
inbreeding depression. Trends Ecol Evol.

[B93] Langschied F, Bordin N, Cosentino S, Fuentes-Palacios D, Glover N, Hiller M, Hu Y, Huerta-Cepas J, Coelho LP, Iwasaki W (2024). Quest for orthologs in the era of biodiversity
genomics. Genome Biol Evol.

[B94] Lasky JR, Josephs EB, Morris GP (2022). Genotype-environment associations to reveal the molecular basis
of environmental adaptation. Plant Cell.

[B95] Lässig M, Mustonen V, Walczak AM (2017). Predicting evolution. Nat Ecol Evol.

[B96] Lee PS, Dong MH, Yan XL, He TY, Yu SF, Wee SL, Wilson J (2023). Blowfly-derived mammal DNA as mammal diversity assessment tool:
Determination of dispersal activity and flight range of tropical
blowflies. Biodivers Data J.

[B97] Leigh DM, Lischer HEL, Guillaume F, Grossen C, Günther T (2021). Disentangling adaptation from drift in bottlenecked and
reintroduced populations of Alpine ibex. Mol Ecol Resour.

[B98] Leroy G, Carroll EL, Bruford MW, DeWoody JA, Strand A, Waits L, Wang J (2018). Next-generation metrics for monitoring genetic erosion within
populations of conservation concern. Evol Appl.

[B99] Lewin HA, Robinson GE, Kress WJ, Baker WJ (2018). Earth BioGenome Project: Sequencing life for the future of
life. Proc Natl Acad Sci U S A.

[B100] Lines R, Juggernauth M, Peverley G, Keating J, Simpson T, Mousavi-Derazmahalleh M, Bunce M, Berry TE, Taysom A, Bernardino AF (2023). A large scale temporal and spatial environmental DNA biodiversity
survey of marine vertebrates in Brazil following the Fundão tailings dam
failure. Mar Environ Res.

[B101] Manu S, Umapathy G (2023). Deep sequencing of extracellular eDNA enables total biodiversity
assessment of ecosystems. Ecol Indic.

[B102] Marshall NT, Vanderploeg HA, Chaganti SR (2021). Environmental (e)RNA advances the reliability of eDNA by
predicting its age. Sci Rep.

[B103] Martins SV, Pilocelli A, Kruschewsky GC, Dias AA, Nabeta FH, Villa PM (2024). Seed bank and aboveground vegetation of Atlantic Forest
re-growing on mining tailings in Mariana: Highlighting diversity patterns of
functional groups. Ecol Res.

[B104] Massey AL, Silva DJF da, Vieira CJ da SP, Allen JM, Canale GR, Bernardo CSS, Bronzoni RV de M, Peres CA, Levi T (2025). Using iDNA to determine impacts of Amazonian deforestation on
Leishmania hosts, vectors, and their interactions. PLoS Negl Trop Dis.

[B105] Mc Cartney AM, Formenti G, Mouton A, De Panis D, Marins LS, Leitão HG, Diedericks G, Kirangwa J, Morselli M, Salces-Ortiz J (2024). The European Reference Genome Atlas: Piloting a decentralised
approach to equitable biodiversity genomics. NPJ Biodivers.

[B106] Miller CV, Bossu CM, Saracco JF, Toews DPL, Rushing CS, Roberto-Charron A, Tremblay JA, Chandler RB, DeSaix MG, Fiss CJ (2023). Genomics-informed conservation units reveal spatial variation in
climate vulnerability in a migratory bird. Mol Ecol.

[B107] Mochales-Riaño G, Fontsere C, Manuel M de, Talavera A, Burriel-Carranza B, Tejero-Cicuéndez H, AlGethami RHM, Shobrak M, Marques-Bonet T, Carranza S (2023). Genomics reveals introgression and purging of deleterious
mutations in the Arabian leopard (Panthera pardus nimr). iScience.

[B108] Murphy WJ, Harris AJ (2024). Toward telomere-to-telomere cat genomes for precision medicine
and conservation biology. Genome Res.

[B109] Nielsen ES, Hanson JO, Carvalho SB, Beger M, Henriques R, Kershaw F, Von Der Heyden S (2023). Molecular ecology meets systematic conservation
planning. Trends Ecol Evol.

[B110] Nigenda-Morales SF, Lin M, Nuñez-Valencia PG, Kyriazis CC, Beichman AC, Robinson JA, Ragsdale AP, Urbán R. J, Archer FI, Viloria-Gómora L (2023). The genomic footprint of whaling and isolation in fin whale
populations. Nat Commun.

[B111] Patin NV, Pitz KJ, Baker JD, Chavez FP, Goodwin KD (2025). Markers or metagenomes: Sequencing marine eukaryotic DNA for
better biodiversity surveys. Metabarcoding Metagenomics.

[B112] Pegueroles C, Pascual M, Carreras C (2024). Going beyond a reference genome in conservation
genomics. Trends Ecol Evol.

[B113] Pereira Lobo F, Benjamim DM, da Silva TTM, de Oliveira MD (2025). Molecular and functional convergences associated with complex
multicellularity in Eukarya. Mol Biol Evol.

[B114] Perry WB, Seymour M, Orsini L, Jâms IB, Milner N, Edwards F, Harvey R, De Bruyn M, Bista I, Walsh K (2024). An integrated spatio-temporal view of riverine biodiversity using
environmental DNA metabarcoding. Nat Commun.

[B115] Peterson Weber JN, Kay EH, Fisher HS, Hoekstra HE (2012). Double Digest RADseq: An inexpensive method for de novo snp
discovery and genotyping in model and non-model species. PLoS One.

[B116] Prince DJ, O’Rourke SM, Thompson TQ, Ali OA, Lyman HS, Saglam IK, Hotaling TJ, Spidle AP, Miller MR (2017). The evolutionary basis of premature migration in Pacific salmon
highlights the utility of genomics for informing
conservation. Sci Adv.

[B117] Quéméré E, Aucourd M, Troispoux V, Brosse S, Murienne J, Covain R, Le Bail P, Olivier J, Tysklind N, Galan M (2021). Unraveling the dietary diversity of Neotropical top predators
using scat DNA metabarcoding: A case study on the elusive Giant
Otter. Environ DNA.

[B118] Quince C, Walker AW, Simpson JT, Loman NJ, Segata N (2017). Shotgun metagenomics, from sampling to analysis. Nat Biotechnol.

[B119] Randin CF, Ashcroft MB, Bolliger J, Cavender-Bares J, Coops NC, Dullinger S, Dirnböck T, Eckert S, Ellis E, Fernández N (2020). Monitoring biodiversity in the Anthropocene using remote sensing
in species distribution models. Remote Sens Environ.

[B120] Rees JS, Castellano S, Andrés AM (2020). The genomics of human local adaptation. Trends Genet.

[B121] Reid BN, Hofmeier J, Crockett H, Fitzpatrick R, Waters R, Fitzpatrick SW (2025). Balancing inbreeding and outbreeding risks to inform
translocations throughout the range of an imperiled darter. Evol Appl.

[B122] Rellstab C, Gugerli F, Eckert AJ, Hancock AM, Holderegger R (2015). A practical guide to environmental association analysis in
landscape genomics. Mol Ecol.

[B123] Rhie A, McCarthy SA, Fedrigo O, Damas J, Formenti G, Koren S, Uliano-Silva M, Chow W, Fungtammasan A, Kim J (2021). Towards complete and error-free genome assemblies of all
vertebrate species. Nature.

[B124] Robinson JA, Räikkönen J, Vucetich LM, Vucetich JA, Peterson RO, Lohmueller KE, Wayne RK (2019). Genomic signatures of extensive inbreeding in Isle Royale wolves,
a population on the threshold of extinction. Sci Adv.

[B125] Rollinson N, Keith DM, Houde ALS, Debes PV, McBride MC, Hutchings JA (2014). Risk assessment of inbreeding and outbreeding depression in a
captive-breeding program. Conserv Biol.

[B126] Romiguier J, Gayral P, Ballenghien M, Bernard A, Cahais V, Chenuil A, Chiari Y, Dernat R, Duret L, Faivre N (2014). Comparative population genomics in animals uncovers the
determinants of genetic diversity. Nature.

[B127] Ruppert KM, Kline RJ, Rahman MS (2019). Past, present, and future perspectives of environmental DNA
(eDNA) metabarcoding: A systematic review in methods, monitoring, and
applications of global eDNA. Glob Ecol Conserv.

[B128] Sales NG, Kaizer MDC, Coscia I, Perkins JC, Highlands A, Boubli JP, Magnusson WE, Da Silva MNF, Benvenuto C, Mcdevitt AD (2020). Assessing the potential of environmental DNA metabarcoding for
monitoring Neotropical mammals: A case study in the Amazon and Atlantic
Forest, Brazil. Mammal Rev.

[B129] Saranholi BH, Rodriguez‐Castro KG, Carvalho CS, Chahad‐Ehlers S, Gestich CC, Andrade SCS, Freitas PD, Galetti PM (2023). Comparing iDNA from mosquitoes and flies to survey mammals in a
semi‐controlled Neotropical area. Mol Ecol Resour.

[B130] Saremi NF, Supple MA, Byrne A, Cahill JA, Coutinho LL, Dalén L, Figueiró HV, Johnson WE, Milne HJ, O’Brien SJ (2019). Puma genomes from North and South America provide insights into
the genomic consequences of inbreeding. Nat Commun.

[B131] Sasso T, Lopes CM, Valentini A, Dejean T, Zamudio KR, Haddad CFB, Martins M (2017). Environmental DNA characterization of amphibian communities in
the Brazilian Atlantic Forest: Potential application for conservation of a
rich and threatened fauna. Biol Conserv.

[B132] Sato JJ (2024). Diets of rodents revealed through DNA
metabarcoding. Mammal Study.

[B133] Savolainen O, Lascoux M, Merilä J (2013). Ecological genomics of local adaptation. Nat Rev Genet.

[B134] Sayers EW, Bolton EE, Brister JR, Canese K, Chan J, Comeau DC, Connor R, Funk K, Kelly C, Kim S (2022). Database resources of the national center for biotechnology
information. Nucleic Acids Res.

[B135] Schneller NM, Strugnell JM, Field MA, Johannesson K, Cooke I (2025). Putting structural variants into practice: The role of
chromosomal inversions in the management of marine
environments. Mol Ecol.

[B136] Secomandi S, Gallo GR, Rossi R, Rodríguez Fernandes C, Jarvis ED, Bonisoli-Alquati A, Gianfranceschi L, Formenti G (2025). Pangenome graphs and their applications in biodiversity
genomics. Nat Genet.

[B137] Segelbacher G, Bosse M, Burger P, Galbusera P, Godoy JA, Helsen P, Hvilsom C, Iacolina L, Kahric A, Manfrin C (2022). New developments in the field of genomic technologies and their
relevance to conservation management. Conserv Genet.

[B138] Shafer ABA, Kardos M (2025). Runs of homozygosity and inferences in wild
populations. Mol Ecol.

[B139] Shafer ABA, Wolf JBW, Alves PC, Bergström L, Bruford MW, Brännström I, Colling G, Dalén L, De Meester L, Ekblom R (2015). Genomics and the challenging translation into conservation
practice. Trends Ecol Evol.

[B140] Shao Y, Zhou L, Li F, Zhao L, Zhang B-L, Shao F, Chen J-W, Chen C-Y, Bi X, Zhuang X-L (2023). Phylogenomic analyses provide insights into primate
evolution. Science.

[B141] Smith SN, Schlupp I, Higgins ED, Watters JL, Bennett K, Bräger S, Siler CD (2022). Development and validation of an environmental DNA protocol to
detect an invasive Caribbean freshwater fish, the guppy (Poecilia reticulata
). Environ DNA.

[B142] Speak SA, Birley T, Bortoluzzi C, Clark MD, Percival-Alwyn L, Morales HE, van Oosterhout C (2024). Genomics-informed captive breeding can reduce inbreeding
depression and the genetic load in zoo populations. Mol Ecol Resour.

[B143] Suarez-Gonzalez A, Lexer C, Cronk QCB (2018). Adaptive introgression: A plant perspective. Biol Lett.

[B144] Szpiech ZA, Xu J, Pemberton TJ, Peng W, Zöllner S, Rosenberg NA, Li JZ (2013). Long runs of homozigosity are enriched for deleterious
variation. Am J Hum Genet.

[B145] Thomsen PF, Kielgast J, Iversen LL, Wiuf C, Rasmussen M, Gilbert MTP, Orlando L, Willerslev E (2012). Monitoring endangered freshwater biodiversity using environmental
DNA. Mol Ecol.

[B146] Thomsen PF, Willerslev E (2015). Environmental DNA - An emerging tool in conservation for
monitoring past and present biodiversity. Biol Conserv.

[B147] van der Valk T, Jensen A, Caillaud D, Guschanski K (2024). Comparative genomic analyses provide new insights into
evolutionary history and conservation genomics of gorillas. BMC Ecol Evol.

[B148] van Oosterhout C (2024). AI-informed conservation genomics. Heredity.

[B149] van Oosterhout C, Supple MA, Morales HE, Birley T, Tatayah V, Jones CG, Whitford HL, Tollington S, Ruhomaun K, Groombridge JJ (2025). Genome engineering in biodiversity conservation and
restoration. Nat Rev Biodivers.

[B150] Vasiliadis M, Freer JJ, Collins MA, Cleary AC (2024). Assessing the trophic ecology of Southern Ocean Myctophidae: the
added value of DNA metabarcoding. Can J Fish Aquat Sci.

[B151] Veilleux HD, Misutka MD, Glover CN (2021). Environmental DNA and environmental RNA: Current and prospective
applications for biological monitoring. Sci Total Environ.

[B152] Vilaça ST, Vidal AF, Pavan ACD, Silva BM, Carvalho CS, Povill C, Luna-Lucena D, Nunes GL, Figueiró HV, Mendes IS (2024). Leveraging genomes to support conservation and bioeconomy
policies in a megadiverse country. Cell Genomics.

[B153] von Seth J, van der Valk T, Lord E, Sigeman H, Olsen R-A, Knapp M, Kardailsky O, Robertson F, Hale M, Houston D (2022). Genomic trajectories of a near-extinction event in the Chatham
Island black robin. BMC Genomics.

[B154] vonHoldt BM, Cahill JA, Fan Z, Gronau I, Robinson J, Pollinger JP, Shapiro B, Wall J, Wayne RK (2016). Whole-genome sequence analysis shows that two endemic species of
North American wolf are admixtures of the coyote and gray
wolf. Sci Adv.

[B155] Wadgymar SM, DeMarche ML, Josephs EB, Sheth SN, Anderson JT (2022). Local adaptation: Causal agents of selection and adaptive trait
divergence. Annu Rev Ecol Evol Syst.

[B156] Walsh G, McMahon BJ, Thörn F, Rödin-Mörch P, Irestedt M, Höglund J (2024). The risk of inbreeding versus outbreeding depression in managing
an endangered and locally adapted population of a sedentary
bird. Conserv Sci Pract.

[B157] Wang J, Santiago E, Caballero A (2016). Prediction and estimation of effective population
size. Heredity.

[B158] Wang Z, Liu X, Liang D, Wang Q, Zhang L, Zhang P (2023). VertU: Universal multilocus primer sets for eDNA metabarcoding of
vertebrate diversity, evaluated by both artificial and natural
cases. Front Ecol Evol.

[B159] Weeks AR, Stoklosa J, Hoffmann AA (2016). Conservation of genetic uniqueness of populations may increase
extinction likelihood of endangered species: The case of Australian
mammals. Front Zool.

[B160] Willi Y, Kristensen TN, Sgrò CM, Weeks AR, Ørsted M, Hoffmann AA (2022). Conservation genetics as a management tool: The five
best-supported paradigms to assist the management of threatened
species. Proc Natl Acad Sci U S A.

[B161] Williams KM, Barkdull M, Fahmy M, Hekkala E, Siddall ME, Kvist S (2020). Caught red handed: iDNA points to wild source for CITES-protected
contraband leeches. Eur J Wildl Res.

[B162] Williams M, Hernandez C, O’Sullivan AM, April J, Regan F, Bernatchez L, Parle‐McDermott A (2021). Comparing CRISPR‐Cas and qPCR eDNA assays for the detection of
Atlantic salmon (Salmo salar L.). Environ DNA.

[B163] Wood SA, Biessy L, Latchford JL, Zaiko A, Von Ammon U, Audrezet F, Cristescu ME, Pochon X (2020). Release and degradation of environmental DNA and RNA in a marine
system. Sci Total Environ.

[B164] Yang Y-Z, Sun P-W, Ke C-Y, Luo M-X, Chang J-T, Chao C-T, Gao R-H, Liao P-C (2025). Towards climate-resilient conservation: Integrating genetics and
environmental factors in determining adaptive units of a xeric
shrub. Glob Ecol Conserv.

[B165] Yates MC, Derry AM, Cristescu ME (2021). Environmental RNA: A Revolution in ecological
resolution?. Trends Ecol Evol.

[B166] Yuan H, Han J, Yang M, Chen S, Pang X (2025). DNA Metabarcoding: Current applications and
challenges. J Agric Food Chem.

[B167] Zhang G, Li C, Li Q, Li B, Larkin DM, Lee C, Storz JF, Antunes A, Greenwold MJ, Meredith RW (2014). Comparative genomics reveals insights into avian genome evolution
and adaptation. Science.

[B168] Zhou Y, Liu M, Yang J (2022). Recovering metagenome-assembled genomes from shotgun metagenomic
sequencing data: Methods, applications, challenges, and
opportunities. Microbiol Res.

[B169] Zoonomia Consortium (2020). A comparative genomics multitool for scientific discovery and
conservation. Nature.

